# 98 Forecasting the Consequences of Collapse in Infection Prevention and Control: A Premortem Analysis Using the Socioecological Model

**DOI:** 10.1017/ash.2026.10520

**Published:** 2026-06-23

**Authors:** Tan Vo, Cameron Lakatos, Jonathan Guerin, Madeline Gardner, Michael Klepser, Minji Sohn

**Affiliations:** 1 Ferris State University College of Pharmacy; 2 Student; 3 Ferris State Universwity College of Pharamcy; 4 Ferris State University

## Abstract

**Background:** Companion animals, including dogs and cats, can harbor pathogenic microorganisms that affect animal health and pose potential zoonotic risks to humans. The U.S. National Action Plan for Combating Antimicrobial Resistance, grounded in the One Health framework, emphasizes collaboration across human, animal, and environment health sectors and calls for improved veterinary oversight of antimicrobial use (AMU). However, contemporary data describing veterinary AMU remain limited, particularly in academic veterinary settings. **Methods:** This cross-sectional retrospective study analyzed encounter data obtained from a Michigan veterinary teaching hospital between June 2022 to May 2025. Companion animal patients were categorized as dogs or cats. De-identified data included demographics, encounter characteristics, service types, reasons for visit, antimicrobial agents, routes of administration, and treatment durations. Only encounters with ≥1 antimicrobial prescription were included. Descriptive analyses were performed, and proportions were reported with 95% Wilson confidence intervals (CIs) where applicable. **Results:** A total of 14,010 encounters, representing 17% (95% CI, 16.8-17.2) of all encounters, involved at least one antimicrobial prescription. These encounters corresponded to 12,401 unique patients, of whom 75.6% were dogs and 24.4% were cats. A lower proportion of dogs were neutered compared with cats (74.5% [95% CI, 73.6-75.4] vs 84.2% [82.9-85.5]). The median patient age was 6.0 years (IQR, 2.2-10.0). Emergency and critical care accounted for 44.4% (95% CI, 43.7-45.1) of antimicrobial-associated encounters, and wellness/follow-up visits were the most common encounter type in both species (38.3% [95% CI, 37.6-39.1]). Compared with dogs, cats more frequently presented for respiratory conditions (10.0% [95% CI, 9.6-10.6] vs 23.5% [22.0-25.0]) and urinary tract disease (9.9% [9.4-10.4]) vs 21.8% [20.4-23.3]). Dogs received more antimicrobial prescriptions per patient than cats (2.49 [95% CI, 2.43-2.56] vs 1.73 [1.60-1.79]), and ≥4 prescriptions per visit were more common in dogs (16.7% [15.9-17.4] vs 4.7% [4.0-5.5]). Amoxicillin-clavulanate accounted for over half of the oral antimicrobial prescriptions in dogs (55.4%, 4,697/8,474) and cats (59.6% [1,608/2,699); cats were associated with a longer treatment of amoxicillin-clavulanate (median 7 days [IQR 7-14] vs 10 days [IQR 7-10]). Fluoroquinolones were the second most common agents in both species, with prolonged duration observed, particularly for pradofloxacin in cats (median 60 days [IQR, 12-60]). **Conclusions:** Antimicrobial prescribing was common among companion animals, with frequent use of broad-spectrum agents and prolonged treatment durations. These findings highlight opportunities for veterinary antimicrobial stewardship, including diagnostic-linked prescribing, duration optimization, and reduction of empiric and repetitive AMU, particularly in outpatient and emergency care settings.

